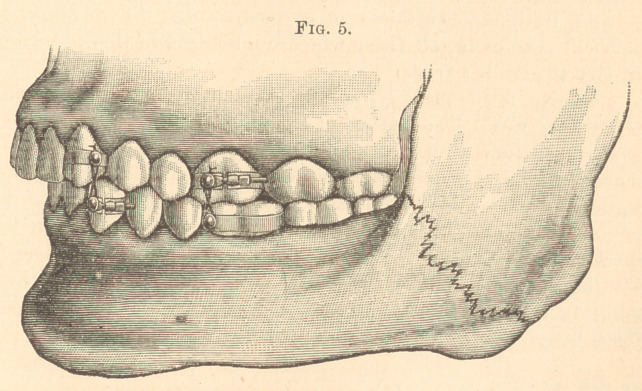# A New Method for the Treatment of Fractures of the Maxillæ

**Published:** 1890-06

**Authors:** Edward H. Angle

**Affiliations:** Minneapolis


### A NEW METHOD FOR THE TREATMENT OF FRACTURES OF THE MAXILLÆ.

BY EDWARD H. ANGLE, D.D.S., MINNEAPOLIS.

MUSCULAR contraction is a most difficult obstacle to overcome in reducing and maintaining in proper apposition most fractured bones. This force, especially in the femur and patella, is so difficult to antagonize that probably the ideal or normal result is never attained.

In fractures of the inferior maxilla it is also a most difficult obstacle in the way of maintaining perfect apposition of the parts, and should always be carefully considered in the construction of an appliance for this purpose. This difficulty is intensified by the large number of movements to which the human jaw is susceptible, and the great number of causes which contribute to their production. That others have recognized the difficulties to be overcome in the treatment of fractures is evinced by the large number of devices and appliances which have been recommended for the purpose.

The methods used by myself in treating fractures of the maxillæ have been so successful and so gratifying that it would seem they approach for efficiency and simplicity more nearly the ideal than any yet devised.

In order that this system of treating fractures of the maxillary bones may be more easily understood, I will divide them into three classes. The first class comprises all simple fractures in which the teeth are good and sufficiently firm in their attachments (especially on each side of the fracture) to afford anchorage for the appliance.

The second class comprises all fractures where the teeth are unsuited, from disease or any other cause, for anchorage, but yet sufficient to give the correct articulation of the jaws.

The third class comprises all fractures where the jaws are edentulous. The following cases treated by myself will enable the reader to comprehend the method peculiar to each class:

Case No. 1 will illustrate Class No. 1. May 29, 1889, Nels Parsons, aged twenty-one, was admitted to the Saint Anthony Hospital of this city. He had fallen from a pile of lumber, a distance of fifteen or twenty feet, and, besides severe bruises, suffered a simple fracture through the symphysis, terminating, however, in front between the central and lateral on the left side, as shown by the line in the engraving, Fig. 1. Upon examination, I found the fractured bone

Fig. 1.

was quite widely separated at the top, and the left central incisor was loosened. The following treatment was adopted: The ends of the fractured bones were placed in their proper position and temporarily fastened by lacing the teeth with silk ligatures.

Bands of very thin German silver were made to encircle and accurately fit the cuspid teeth. A small tube of German silver, one-half inch in length, was soldered to each band and in exact alignment; a piece of wire accurately fitting the bore of these tubes, bent at right angles at one end and having a screw cut upon the other end, was slipped through each tube and secured therein by adjusting a nut on the screw. The bands were cemented in position upon the teeth by means of oxyphosphate cement, as shown in Fig. 1.

After the cement had become thoroughly set, the nut was then tightened until the fractured ends of the bone were drawn snugly together.

The appliance was worn without displacement or trouble for twenty-one days, when it was removed, the bone having become firmly united. I may add that, during the time the appliance was worn, so firmly was the jaw supported, the patient suffered little if any inconvenience, and after the third day partook regularly of his meals, using his jaw freely, but of course avoided the hardest particles of food. After removing the appliance a careful impression of the jaw was taken, a model made, and the appliance transferred to the model, exactly as shown in the engraving. The lower part of the jaw is, of course, diagrammatic, and was added by the engraver to show the line of fracture.

It should be borne in mind that the principle upon which this appliance is based is not the same as when the teeth are simply wired together, but very different; for, in wiring, the upper parts of the fracture only are tipped or drawn together, and no pressure or support is given to the lower parts; while in the method here shown it will be seen that, by reason of the bands and pipes being rigidly attached to the anchor teeth, tipping is impossible, and pressure is exerted equally upon both parts (upper and lower) of the fracture as they are drawn together by the screw; or, as my friend, Dr. Charles G. Brown (who first suggested to me this use of the screw), puts it, "It is a Malgaigne splint, if you please, except that the hooks are not foreign bodies."

FIG. 2.[1]

This device may be applied in any locality in either jaw, provided suitable teeth for anchorage be not too remote from line of fracture. The screw may be bent to accommodate the curve in the arch, should the fracture occur in the region of the cuspid.

The treatment for cases of the second class is illustrated in Case No. 2. On July 4, 1889, William Fraley, aged forty-five, was ad-

[1] These bands, tubes, wires, screws, and nuts are some of the appliances known as "Angle's Regulating and Retaining Appliances," devised and used for the purpose of correcting irregularities of the teeth. They may be made by any ingenious dentist, or procured from any dealer in dental goods.

mitted to the Minneapolis City Hospital. A blow from a policeman's club had produced two simple fractures of the inferior maxilla. The first was an oblique fracture of the right side, beginning with the socket of the second bicuspid, extending downward and backward, involving the socket of the first molar, breaking out the second bicuspid and greatly loosening the first molar. The second molar had been lost years before, while the third, as well as the remaining teeth, were much abraded and loosened by salivary calculus, thus making the application of the appliance described in Case No. 1 impossible. The second fracture was situated on the opposite side, high up in the ramus. Because of swollen condition of the parts, I could not detect the exact line of fracture, but the grinding of the ends of the bone and the great pain occasioned thereby were unmistakable evidences of a fracture. The patient, as in all such cases, was unable to close the jaws. The fracture on the right side was widely separated, and the anterior piece much depressed by reason of the contraction of the depressor muscles, while the posterior piece of bone was drawn firmly up, the molar

Fig. 3.

teeth occluding. (See Fig. 3.) The following treatment was used: Bands were made to encircle all four of the cuspid teeth, they being the most firmly attached in their sockets. The fractured ends of the bones were placed in apposition, the lower jaw closed carefully. The occlusion of the lower teeth upon the upper required so considerable force and occasioned so much pain that it became necessary to anæsthetize the patient. Points on the bands for the necessary attachments were carefully noted. The bands were then slipped off the teeth, and little pipes (shown at *c*, Fig. 2) soldered at the necessary points, after which the bands were cemented in their proper positions upon the teeth, and two small traction screw-wires, the same as shown at *b*, Fig. 2, were slipped into the pipes. The jaws were closed and the nuts tightened on the screws, until the jaws were drawn firmly together, and each tooth occupied its exact position in occluding upon its fellow of the opposite jaw. Both fractures were then carefully examined and found to be in perfect apposition, and presented the appearance shown in Fig. 3. The most natural position for the jaw and the muscles had been secured, thus placing the parts in their natural position of relaxation and rest.

During an attack of coughing in the night following, one of the bands was wrenched loose, but was replaced the next day without trouble. No further accidents occurred. The patient readily took nourishment through the spaces between the teeth. Thus the fractured jaw was firmly supported without the least motion for twenty-two days, when the appliance was removed, showing most excellent results.

The patient was a great lover of the clay pipe, which accounts for the much-worn condition of the incisors.

The following case possesses several points of special interest, although the fractures occurred in regions similar to the case just described, and the appliances, though involving similar mechanical principles, will be found to be greatly simplified.

December 28, Thomas Brennan was admitted to the Dental Infirmary of the University of Minnesota, suffering from the effects of a blow received on the left side of the jaw from a cant hook while working in a lumber camp in Wisconsin, which produced fracture of the jaw in two places. The first fracture was on the left side, beginning between the first and second bicuspids, and extending downward and backward, and involving the lower part of the anterior root of the first molar. The second fracture was on the right side directly through the angle of the jaw. The fractures had occurred thirty-two days previous to his admission to the infirmary, during which time nothing had been done to reduce them. He reported that he had called upon a physician, who supposed the trouble was merely an abscessed tooth, and had lanced the gum with the view of reducing the swelling. Later the patient had called upon a dentist in one of the smaller towns, who also failed to diagnosticate the fracture, and extracted both bicuspids in the hope of giving relief.

Upon examination, I found considerable swelling in the region of this fracture, with the usual result: the patient being unable to close his mouth by reason of the anterior piece of the fractured bone being drawn down by the contraction of the depressor muscles. A false joint had also become established, and the bones could be easily worked without causing pain.

At the point of fracture, on the right side, there was little or no displacement; the swelling was also slight. With the assistance of

FIG. 4.

Professor Leonard the patient was anæsthetized; the ends of the bones were then rubbed forcibly together with the view of breaking up the false attachments and stimulating activity in repair.

The ends of the bones were now placed in perfect apposition, and the jaw closed, taking great care to articulate the teeth in their correct position against the upper ones.

The jaw was now firmly bound in this position to the upper teeth, in the same manner as described in Case No. 2, with this

FIG. 5.

difference, that the method was improved upon and simplified by using clasp bands, as shown in Fig. 4.

No cement was used, and, instead of the screws, small metallic buttons were soldered to the sides of the bands (as shown in the cut), around which fine binding wire was wrapt in the form of a figure 8. (See Fig. 5.)

The bands seen upon the molar teeth in the engraving were not used in this case, but are shown for the purpose of illustrating how they may be used in cases of comminuted fracture. At the end of seventeen days the bands were removed and the patient discharged, the bone having been firmly united.

It might be urged as an argument against this method that, the teeth being closed and the jaws being firmly bound together, the patient would be unable to take sufficient nourishment. It, however, rarely happens that a patient is found without some teeth missing, thereby leaving abundance of space for the passage of the liquid foods, and even if all the teeth are sound and in perfect position, it has been proved that there is plenty of space between the teeth and behind the molars and between the upper and lower incisors for taking all nourishment necessary. In such cases more time would be consumed in taking nourishment, but this obstacle is compensated for by the main points of advantage in its favor, such as cleanliness and greater comfort to the patient, as compared with the many bulky and awkward appliances in use.

Third, its extreme simplicity enables any one with ordinary mechanical ability, when provided with a set of clamp bands, to easily and quickly set all ordinary cases of fracture.

And, lastly, the certainty of correct results will, I think, be sufficient reasons for all those who are interested in this branch of surgery to give it a trial.

Class No. 3, comprising fractures of edentulous jaws, are fortunately very rare. The method of treatment I propose is similar in principle to that already described in Class No. 1, except that in place of the teeth small bone hooks are used, drilling for their reception a suitable cavity on each side of the fracture comparing in position to the original sockets of the teeth, the same as if the operation of implanting teeth was intended; the cavities thus made need not be nearly so large or deep. They should also be drilled obliquely to correspond to the course taken by the hooks. The hooks before insertion should of course be made antiseptic.

## Figures and Tables

**Fig. 1. f1:**
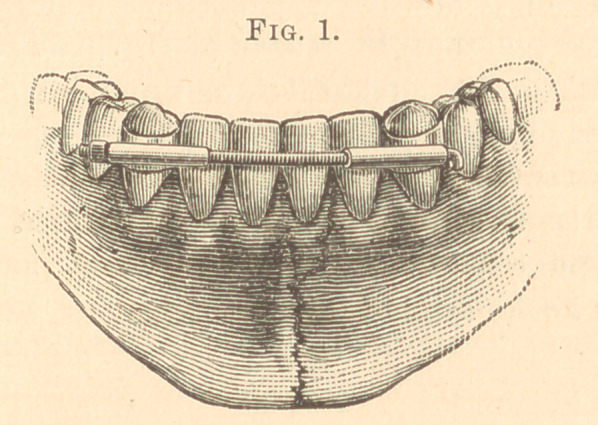


**Fig. 2. f2:**
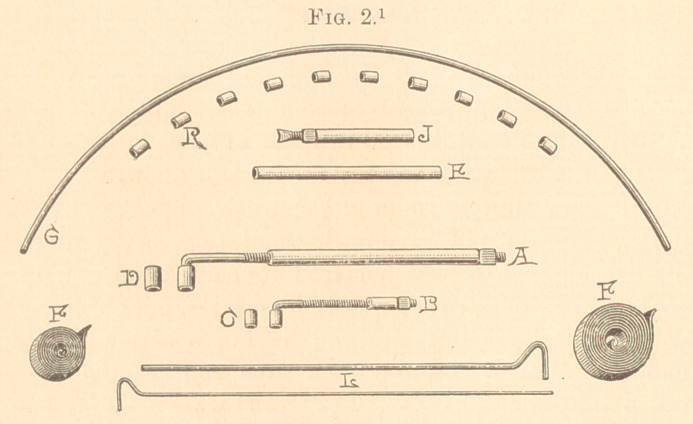


**Fig. 3. f3:**
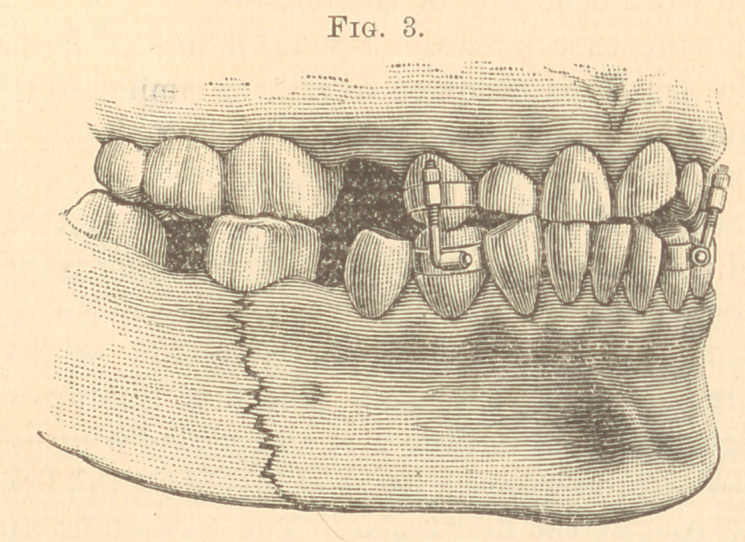


**Fig. 4. f4:**
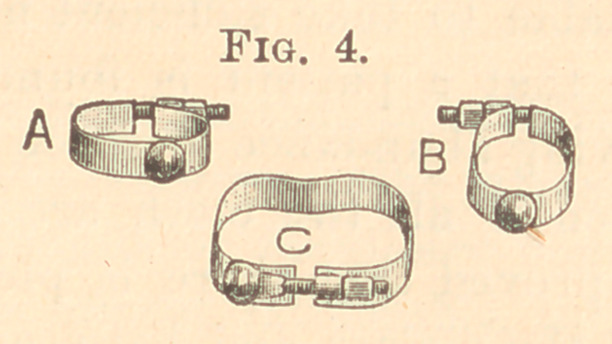


**Fig. 5. f5:**